# Supplementary material for: High-content stimulated Raman histology of human breast cancer
Source: Theranostics. 2024 Jan 27;14(4):1361–70. doi: 10.7150/thno.90336 (PMC10879861; doi:10.7150/thno.90336)
Supplement: Supplementary file 1 — Supplementary figures and table. [file thnov14p1361s1.pdf]

Supplementary Materials

High-content Stimulated Raman histology of breast cancer

Hongli Ni,[1,#] Chinmayee Prabhu Dessai,[2,#] Haonan Lin,[1,#] Wei Wang,[3] Shaoxiong Chen,[4] Yuhao Yuan,[1] Xiaowei Ge,[1] Jianpeng Ao,[1] Nolan Vild,[1] and Ji-Xin Cheng[1,2]

[1]Department of Electrical and Computer Engineering, Boston University, 8 St. Mary's St., Boston, MA, 02215, USA

[2]Department of Biomedical Engineering, Boston University, 44 Cummington Mall, MA 02215, USA

[3]Hologic Inc., 250 campus drive, Marlborough, MA 01752

[4]Indiana University School of Medicine 340 West 10th Street, Fairbanks Hall, Suite 6200, IN 46202

[5]These authors contributed equally.

1. Selective sampling result with different training dataset

   The RFE selection can vary with different training sets. As shown in Figure S1, there is one channel difference between the selection results trained on sample 1 and on sample 2. Both RFE training used 2100 pixels selected from the whole FOV as described in the main context 2.3. Both the spectral selections give good HC-SRH results with high SSIM and PSNR.

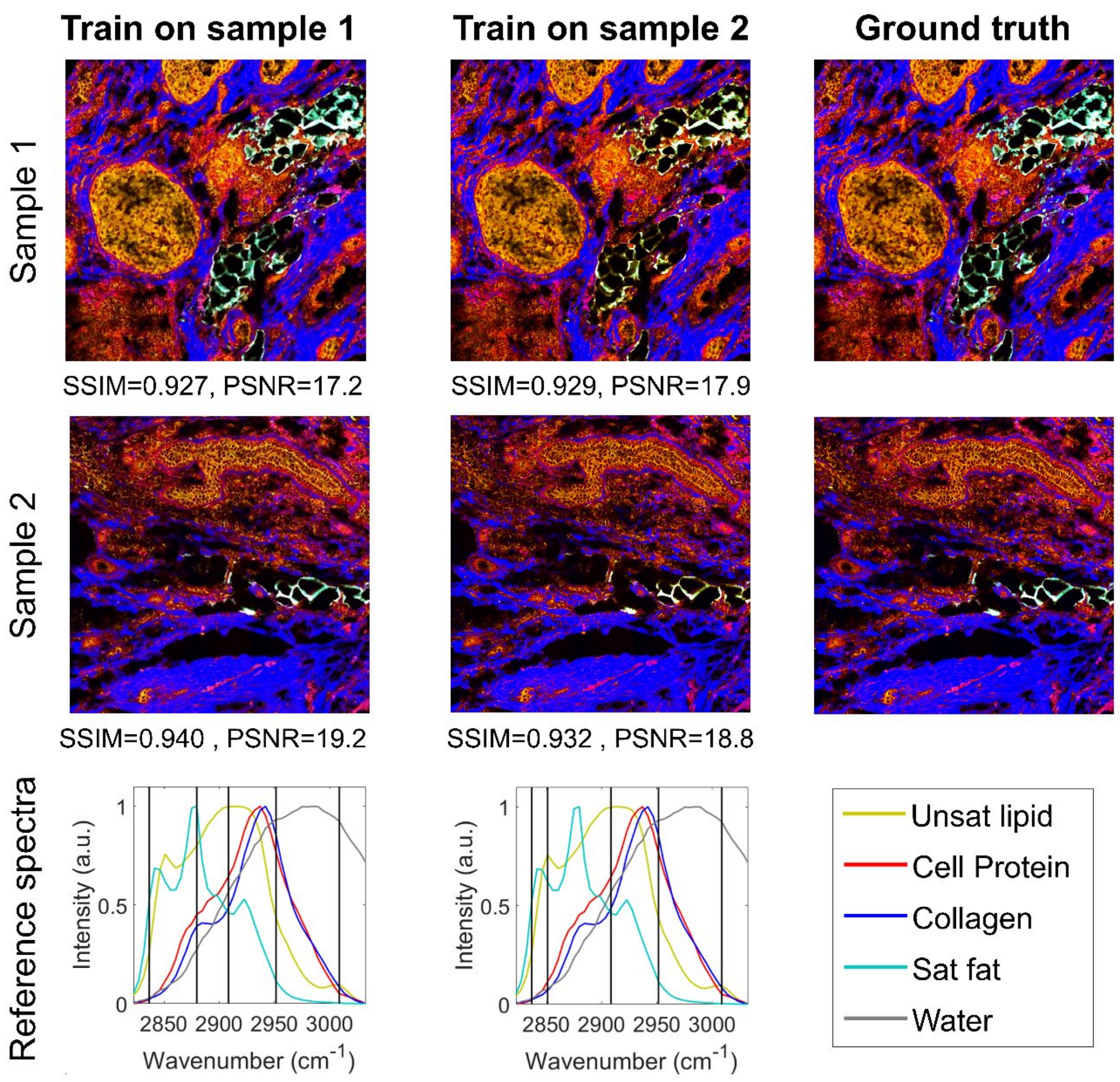

Figure S1. Comparison of HC-SRH result with different RFE selections. FOV: 624X627 μm. SSIM: structural similarity. PSNR: peak signal-to-noise ratio.

2. Peak registration for fingerprint and C-H SRS imaging of breast cancer

Peak	Cytoplasm	Cell nuclei	ECM	Fat cell	Registration
785		X			DNA (O-P-O)
825~830		X			DNA (O-P-O stretch)
855	X	X			Tyrosine
855			X		Collagen
880	X			X	Lipids/carbohydrates
880			X		Collagen

890				X	Solid state TAGs (CH3 rocking vibrations)
920	X	X	X	X	Proline ring / glucose / lactic acid (C–C stretch)
940			X		C–C stretch backbone
1005	X	X		X	Phenylalanine / carotenoids
1005			X		Phenylalanine
1035	X	X	X		Phenylalanine (C–H in-plane bending mode)
1065				X	Skeletal C–C stretch lipids
1075	X	X			Lipid (C–C or C–O stretch) / Nucleic acids (PO2 stretch)
1095	X	X			Phosphodiesters (O-P-O)
1100				X	Stearic acid
1130					Lipids (C-C stretch)
1205	X	X			Hydoxyproline / tyrosine
1250			X		Collagen (CH2 wag, C–N stretch)
1260	X	X			Protein (Amide III)
1300				X	Lipids (CH2 deformation)
1305		X			Adenine, cytosine
1455	X	X	X	X	Proteins/elastin/collagen/phospholipids (CH2)
1490		X			Nucleic acid purine bases
1585		X			Nucleic acid (Pyrimidine ring)
1640~1670	X	X	X		Protein (Amide I)
1660	X			X	Lipids (C=C)
1730 & 1746				X	Ester (C=O. polymorphic form)
2850					Lipids (symmetric CH2)

2885					Asymmetric CH2 stretch of lipids
2925					Saturated bonds of lipids
2930	X	X		X	Lipids (asymmetric CH2 stretch)
2940	X	X	X		Protein (C-H stretching)
3010	X				Unsaturated Lipids (=CH)

Table S1. Peak registration for fingerprint and C-H SRS imaging of breast cancer.

3. Verification of lipid distribution mapped by HC-SRH

We performed a further validation of our chemical mapping results with Nile red staining. Nile red is a standard staining for intracellular lipids. We imaged a fresh-frozen human invasive ductal carcinoma sample with HC-SRH following the procedure described in the main text. After that, we stained the sample with 10 μM Nile red solution and then acquired two-photon image of the sample for the validation. The results are shown in Figure. R1, where the unsaturated lipid mapped by HC-SRH matches well with the Nile red two-photon fluorescence result. The difference between the Nile red result and HC-SRH result should be due to the presence of some hydrophobic proteins that also give fluorescence with high-concentration Nile red staining [1]. With this experiment, we verified that the lipid mapped by HC-SRH is correct.

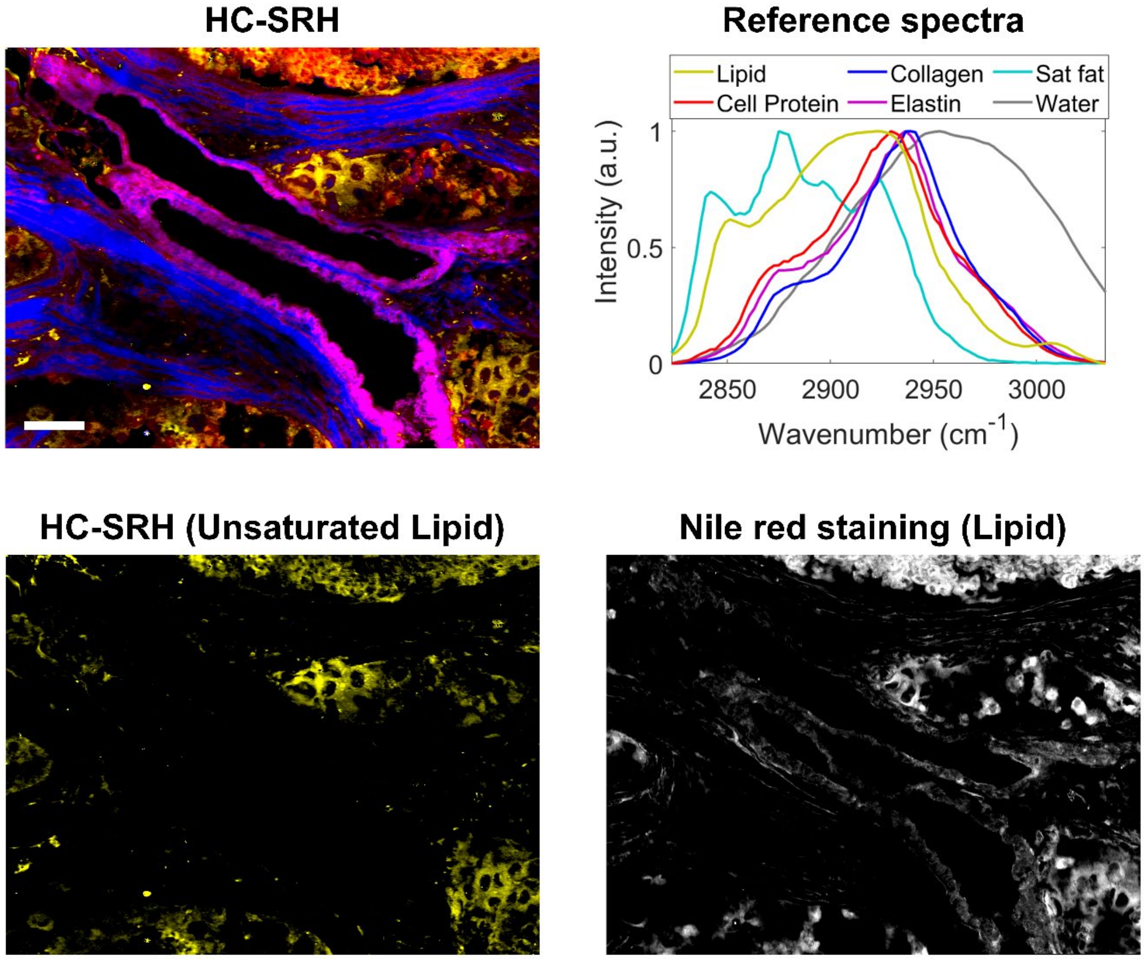

Figure S2. Validation of lipid map of HC-SRH with Nile red staining. Scale bar: 50 μm.

4. Mapping collagen and elastin with C-H HC-SRH

We explored the capability of our method in differentiating collagen and elastin by imaging a fresh-frozen human breast cancer sample in a field of view containing cancer-adjacent vessels. The vessel wall contains abundant elastin and collagen, and thus is a good test bed for exploring the HC-SRH capability in separating elastin and collagen. The ground truth for collagen and elastin distribution is obtained by second harmonic generation and two-photon auto-fluorescence, respectively. The reference spectra of collagen and elastin is obtained from a collagen-rich area and an elastin-rich area in the human breast cancer sample. As shown in Figure. 2R, HC-SRH well separates the collagen with elastin based on their subtle spectral difference. The collage and elastin mapped by HC-SRH have good correlation with the ground-truth second harmonic generation and two-photon auto-fluorescence result. We observed a small crosstalk in between the collagen and elastin channel in HC-SRH, which is because the spectra of the two components are too similar. The cross talk should be alleviated by adding the fingerprint information.

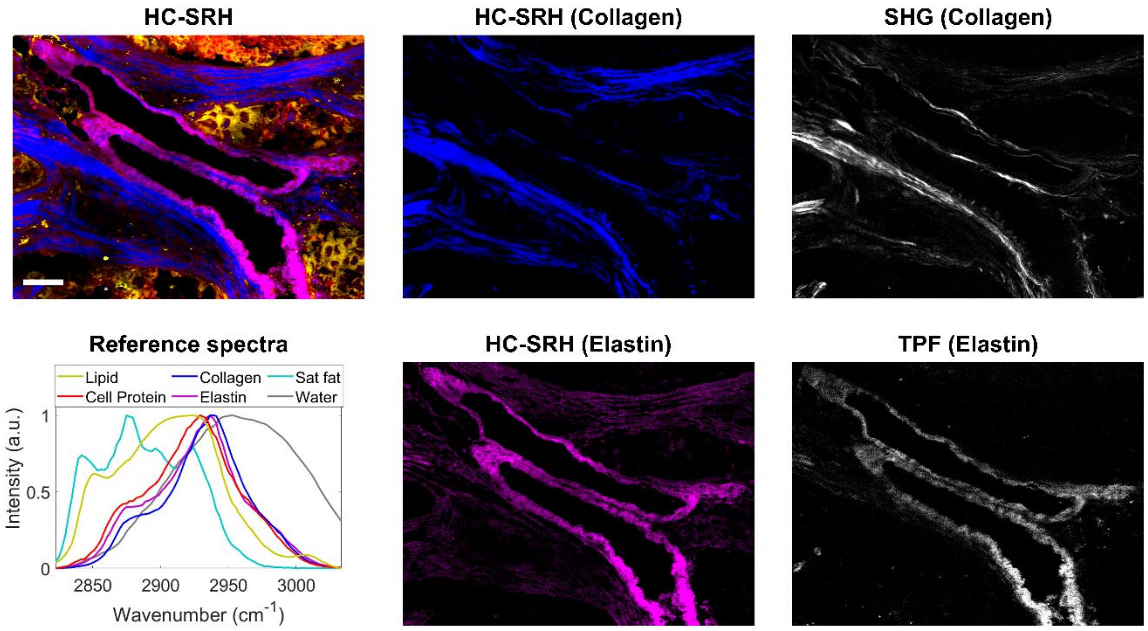

Figure S3. Mapping collagen and elastin with HC-SRH. Scale bar: 50 μm.

Reference:

1. Greenspan P, Fowler SD. Spectrofluorometric studies of the lipid probe, nile red. Journal of Lipid Research. 1985; 26: 781-9.
